# Hospital Expenditure—The Commissariat

**Published:** 1897-02-06

**Authors:** 


### The Institutional Workshop.

### HOSPITAL EXPENDITURE—THE COMMISSARIAT.

### XXXV.—SPECIMEN FORMS (*continued*).

It has been seen that in forming an estimate of the food consumed by patients in a hospital, it is necessary to check the quantities of each article used day by day. Every day the numbers vary and the diets vary, so that a weekly record would be only partially accurate. It will not, however, be found necessary to form a daily return of this nature for other members of the hospital household. The numbers vary little and the bill of fare remains substantially the same from day to day, so that a weekly return will answer all purposes. In attempting to gauge the exact consumption of each article by the household, it becomes at once apparent that the provisions used fall under two headings.

There are first, as in the case of the patients, articles such as meat, vegetables, and milk, which must be ordered in daily, and for which it is necessary that the housekeeper should make an estimate of probable requirements on the previous day. Before making this estimate, which must necessarily be in the hands of the secretary early in the afternoon, the housekeeper will have examined what remains over from dinner towards the next day's needs, and will frame her orders to cover the requirements of breakfast, dinner, and supper. We append a form which may be found useful for this purpose. Allowance has been made for three distinct tables. There are often five or six in even moderate-sized institutions, as when the matron is separately served, the dispensers and assistants have a table to themselves, and men and women servants have separate meals. There are, of course, too, in every hospital distinct meals for day and night nurses. But these distinctions need not be observed in estimating the quantities required, and in small hospitals, where little difference is made in the fare provided at the separate tables, one heading comprehending the amount required for the whole household will prove sufficient.

These daily order forms or estimates will frame a complete record so far as they go of the food consumption of the household, either as a whole or in each separate department. But only certain articles have been enumerated. There are many other items which are ordered in large quantities weekly, monthly, or quarterly, according to the amount of store-room accommodation, and on which the matron can draw at will. It is the use of these articles that it is most difficult to check, and though the danger of waste is less than with perishable provisions, all housekeepers are aware of the insidious manner in which stores run out before their time, and of the difficulty often experienced in accounting for variations in the rate of consumption. It is the duty of the secretary or manager of the institution to see that such articles as tea, sugar, seasonings, &c., are kept always in stock. The duty of the matron begins with the reception of the goods, and are not completed till she has rendered an account of every ounce of each item in her grocery list. There can be no doubt that well-ordered premises exercise an important influence in promoting economy, and that the provincial matron who has at her command a convenient room for receiving and weighing all provisions, before despatching them to tiled larders or cool, well-lighted store-rooms, has a great advantage over her London compeer who may be compelled to find room for an infinity of heterogeneous articles in some gas-lighted cupboard under ground. But, however poor the store-room accommodation may be, it is certain that some articles will have to be kept in stock, and the process of regulating the stores is the same be they few or many.

Form A.—Order Form for Household.

| Number. | Medical Staff. | | Nurses' Table. | | Servants. | | Total. |
|---|---|---|---|---|---|---|---|
| | lbs. | Description. | lbs. | Description. | lbs. | Description. | |
| Meat Joints.. | | | | | | | |
| ,, for Soup | | | | | | | |
| ,, Various. | | | | | | | |
| Fish ... ... | | | | | | | |
| Poultry ... | | | | | | | |
| Potatoes ... | | | | | | | |
| Vegetables ... | | | | | | | |
| Fruit ... ... | | | | | | | |
| Eggs ... ... | | | | | | | |
| Milk ... ... | | | | | | | |
| Butter ... ... | | | | | | | |
| Bread ... ... | | | | | | | |
| Cheese... ... | | | | | | | |
| Bacon ... ... | | | | | | | |

Date......................

Sister....................... ......

All articles received must, in the first place, be weighed or counted on their arrival, and the exact amount received must be recorded, with the date, in a book kept for that purpose in the store-room. The housekeeper must take nothing for granted. She must not be reduced to asking the secretary, "Did you order the same amount as usual last month?" when her stores run out; she must know for certain all that she has received, and be able to verify her calculations by instant reference to her books.

In order to secure a weekly return of all the articles used, it will be necessary for the housekeeper to be provided with a book of foils and counterfoils on which all transactions with the store-room can be recorded. She may not herself have time personally to superintend the giving out of all stores, but whether this be so or not, no article should ever be issued, either by herself or her delegate, except a foil noting the amount issued be filed at the moment, and a corresponding note be made in the housekeeper's own book of counterfoils.

A rough indication of such a store-room order book is here given. The orders should be prepared in long, narrow-shaped books, perforated for more ready use:—

| | |
|---|---|
| Tea. | Tea. |
| ................................ | ................................ |
| Date ........................... | Signed ........................ |
| Moist Sugar. | Moist Sugar. |
| ................................ | ................................ |
| Date ........................... | Signed ......... .............. |
| Rice. | Rice. |
| ................................ | ................................ |
| Date ........................... | Signed ........................ |
| &c. | &c. |

The weight or quantity of each article issued will be entered on both foil and counterfoil. The date is inserted on the counterfoil retained in the book, and from these counterfoils recording the use of every article may be prepared without difficulty, forms showing the weekly consumption of all stores. The signature, which may be merely initials, is written on the foil, and is the authority, without which no stores should ever be issued, unless the establishment admits of the matron herself retaining sole direction of the store-room. These foils, pricked on separate files, must be periodically checked to see that no waste occurs, and compared, if necessary, with the counterfoil in the matron's book. Thus, if the stores run out before their time, it is possible to fix the exact week, perhaps the exact days on which more than usual was required, and ascertain the cause to the avoidance of all possibity of mistake. A check is also placed on that fertile source of waste in every store-room, the careless and inexact measurement or weighing of quantities. For it will become clear that if 50 lbs. of tea were weighed and found correct at the beginning of the quarter, and all comes to an end when but 47 or 48 lbs. have been issued, a daily leak in the measurement of the allowance has been in progress, and when this has been once ascertained it is easily checked. Stores issued for patients' use may be entered by the matron in a separate book of counterfoils, coloured differently, to afford a ready method of distinction.

The matron's weekly return of the quantities used of groceries, and other dry goods, combined with Form A issued daily, will furnish the data for a return showing the average weekly, quarterly, and yearly consumption of all articles of food by members of the household. To estimate the average cost of the food of each inmate of the institution is now a mere matter of detail, worked out by reference to accounts kept by every manager. The distinction between the cost of nurses and servants, if any exists, is easily reckoned by striking out from the cost of the latter such articles as are not supplied to their table, such as salads or fresh eggs.

If a large number of the medical staff is boarded a separate dailyestimate should be furnished of their bill of fare; and, again, all orders of groceries, &c., for their use should be entered on separately tinted foils, the same plan being adopted as for the household. Their requirements and hours of meals being commonly quite different to those of other members of the household, this arrangement will be found not only more accurate but far more convenient and economical.

